# Transcalvarial Cutaneous Squamous Cell Carcinoma With Dural Invasion: Social Determinants as Drivers of Advanced Disease

**DOI:** 10.7759/cureus.97188

**Published:** 2025-11-18

**Authors:** Camille Gorena, Borna Amir-Kabirian, Ben Xie, Vivek Sharma

**Affiliations:** 1 Dermatology, University of Louisville, Louisville, USA; 2 Hematology and Medical Oncology, University of Louisville, Louisville, USA; 3 Oncology, University of Louisville, Louisville, USA; 4 Hematology and Medical Oncology, University of Louisville School of Medicine, Louisville, USA

**Keywords:** immunosuppresion, non-melanoma skin cancer, scalp neoplasms, social determinants of health, squamous cell carcinoma

## Abstract

Cutaneous squamous cell carcinoma (cSCC) of the scalp is a common malignancy, particularly among older individuals with prolonged sun exposure. It is usually superficial and easy to treat with local interventions such as surgery and topical agents. In rare cases, however, untreated or advanced lesions can invade deep structures such as the calvarium, dura mater, or brain parenchyma, requiring multidisciplinary intervention. Immunosuppression from any cause, including untreated human immunodeficiency virus (HIV), significantly increases both the incidence and severity of cSCC, particularly in marginalized populations.

We report a case of a 60-year-old, unhoused Caucasian male from the Sun Belt region with a history of malnutrition and longstanding, untreated HIV infection. He presented with a large, ulcerated frontoparietal scalp mass measuring 8.6 x 10.5 x 8 cm (anteroposterior × transverse × craniocaudal). Magnetic resonance imaging (MRI) of the brain revealed the mass extended through the inner table of bone into the epidural space, resulting in diffuse meningeal enhancement. Following a multi-disciplinary discussion, he underwent a complex craniotomy with bone flap removal, requiring collaborative intervention from neurosurgery, plastic surgery, and otolaryngology. After extensive surgical excision of the lesion, reconstruction was achieved using a latissimus dorsi flap, providing adequate soft tissue coverage and restoring the integrity of the scalp.

This case highlights the aggressive behavior of cSCC in immunocompromised patients and the potential for extensive local invasion in the setting of delayed dermatological care. Public health interventions for the unhoused population, such as skin cancer screenings, sunscreen distribution, and support for antiretroviral therapy adherence in HIV-positive individuals, can play a critical role in reducing health disparities. Promoting early detection and prevention of skin cancer by increasing access to care can lead to improvements in overall health outcomes in this vulnerable group.

## Introduction

Cutaneous squamous cell carcinoma (cSCC) is a common cutaneous malignancy with increased prevalence in older patients with prolonged sun exposure. In the United States, the estimated incidence exceeds one million cases annually, with metastatic rates of approximately 4% [1]. While the majority of cSCC cases demonstrate favorable outcomes with early detection and appropriate treatment, delayed diagnosis or inadequate intervention can allow for progression to locally advanced or metastatic disease [1]. Lesions of the scalp are of particular concern due to proximity to critical structures. In aggressive cases, cSCC of the scalp has potential for invasion into the skull, meninges, and brain parenchyma. Although rare, such an invasion has the potential to cause neurological complications and death and is associated with a poor prognosis requiring multidisciplinary treatment [2,3].

Several risk factors have been well-established for the development of aggressive cSCC, including chronic UV exposure, advanced age, fair skin phenotype, and immunosuppression [1]. Among the immunosuppressed, patients with human immunodeficiency virus (HIV) demonstrate an increased incidence of cSCC. Cohort studies report a two to three-fold higher incidence compared with HIV-negative patients, with risk increasing as CD4 counts fall [4].

The unhoused population experiences a compounding of multiple cSCC risk factors, including increased HIV prevalence. This vulnerable demographic is disproportionately affected by adverse social determinants of health, such as food insecurity, leading to nutritional deficiencies, limited access to healthcare, increased sun exposure, and heightened risk of exposure to infectious diseases. Due to these factors, advanced-stage head and neck cancers are more likely to be seen among the unhoused population [5,6].

We present a case of extraordinarily advanced cSCC in an immunocompromised patient experiencing homelessness, demonstrating transcalvarial invasion with dural involvement. This case exemplifies the devastating consequences of delayed diagnosis in high-risk populations and underscores the importance of enhanced surveillance and prompt therapeutic intervention to prevent mortality in patients with scalp cSCC.

## Case presentation

A 60-year-old Caucasian male from the Sun Belt region presented with a large frontoparietal scalp mass and a medical history significant for latent syphilis, HIV, malnutrition, and documented medication non-adherence. The patient reported that the lesion appeared approximately 10 years prior as a pruritic, mildly tender erythematous papule. Lesion progression accelerated following traumatic impact with a metal structure, which precipitated the development of malodorous discharge. In the subsequent months, the scalp lesion had undergone significant expansion to about the size of a tennis ball.

The patient’s social history was significant for housing instability, with clinical manifestations of malnutrition and muscle wasting. He denied alcohol use, tobacco use, and drug use. He was diagnosed with HIV in 2003 following high-risk sexual behaviors, including unprotected receptive anal intercourse with male partners. His laboratory results at that time revealed a CD4+ count of 132 cells/mm³ and an HIV viral load of 173,000 copies/mL. Despite initiation of antiretroviral therapy and antibiotics at that time, the patient reported inconsistent adherence due to medication theft, related to a lack of secure storage.

**Figure 1:**
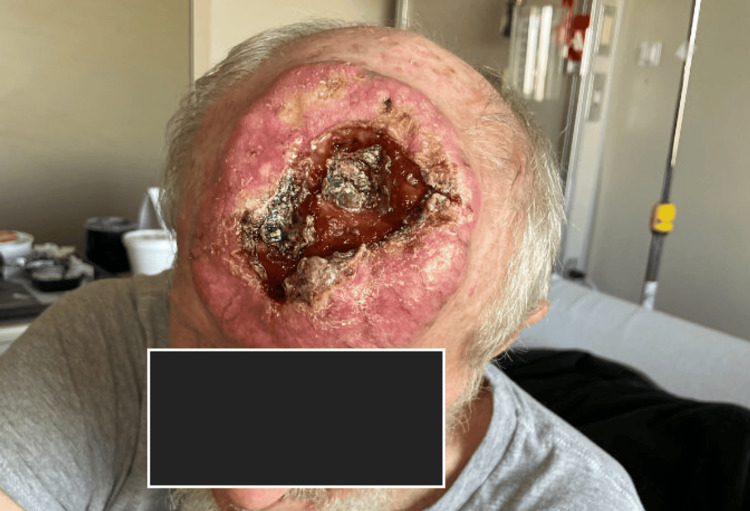
Initial presentation of the fronto-parietal mass

**Figure 2:**
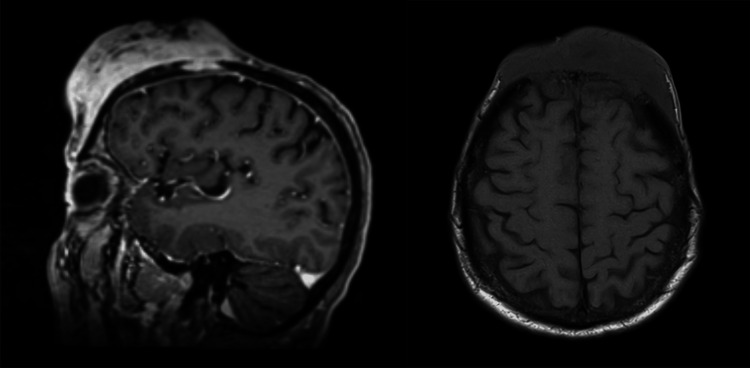
Sagittal and axial MRI views of the brain depicting a large fronto-parietal scalp mass with skull invasion and dural involvement

Physical examination revealed a large, exophytic scalp mass measuring 8.6 x 10.5 x 8 cm (anteroposterior × transverse × craniocaudal) (Figure 1). Histopathological examination confirmed the diagnosis of cutaneous squamous cell carcinoma of the scalp. Wound cultures were positive for the growth of *Pseudomonas aeruginosa*, *Serratia marcescens*, and methicillin-resistant *Staphylococcus aureus* (MRSA). The patient underwent magnetic resonance imaging (MRI) of the brain and computed tomography (CT) of the head (Figure 2). Imaging demonstrated an 8 cm homogeneously enhancing, ulcerated frontal scalp mass with transcalvarial extension through the inner cortical table of bone and epidural space, creating a large 4.8 cm deep frontal bony defect. The cSCC was staged as pT4b.

**Figure 3:**
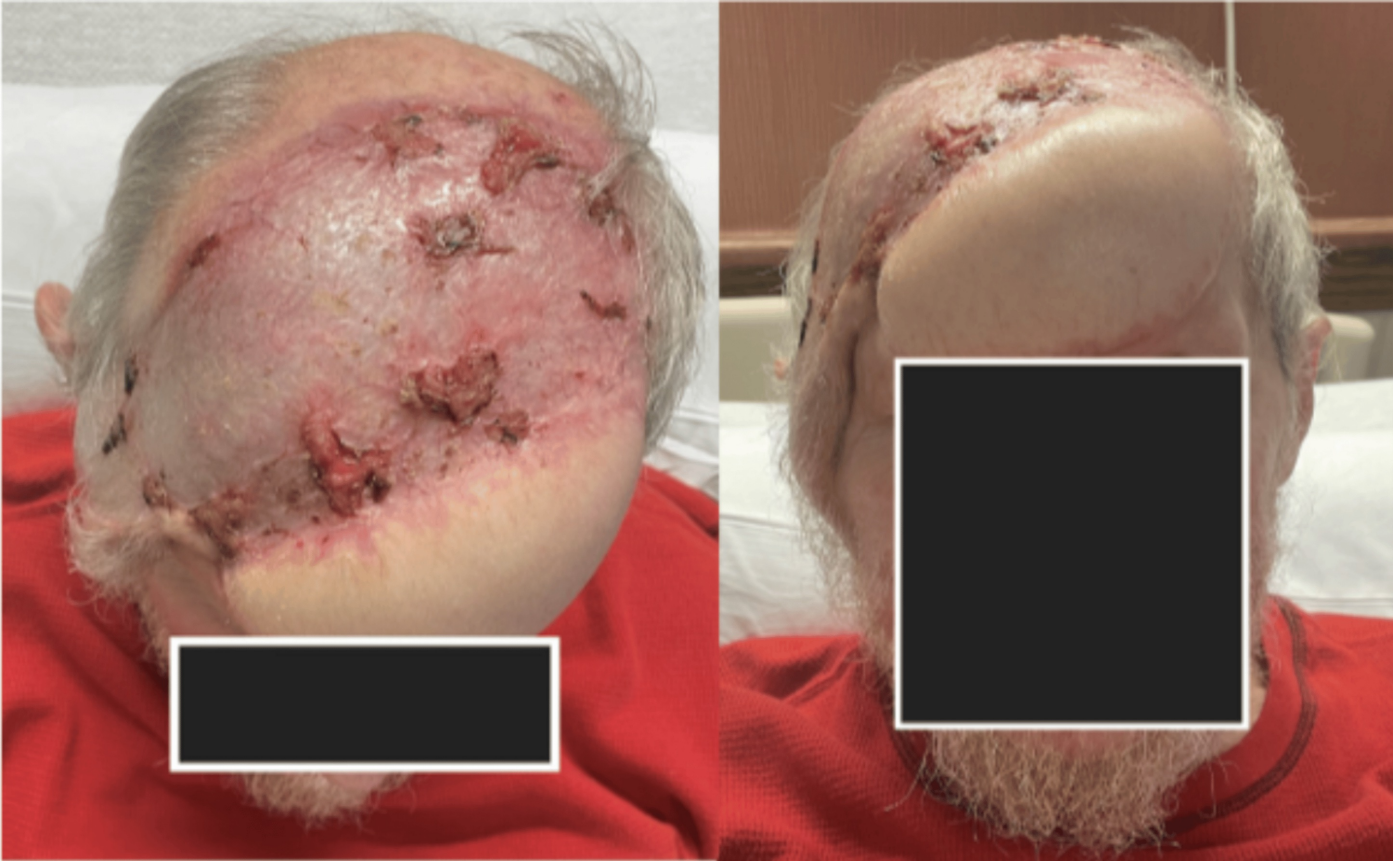
Reconstruction of the scalp defect with a split-thickness skin graft

**Figure 4:**
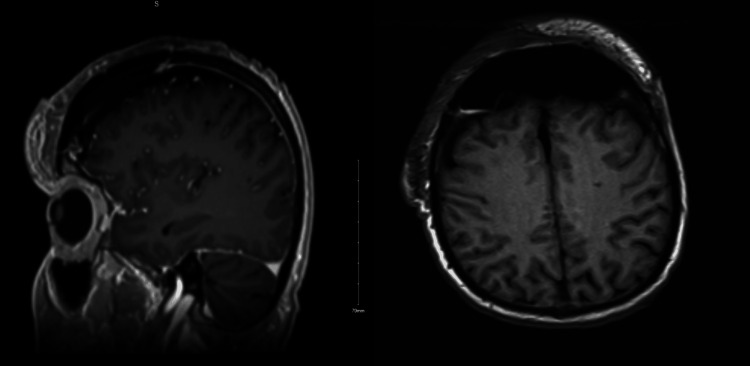
Sagittal and axial MRI views of the brain showing resection of the fronto-parietal mass, placement of a craniplasty graft, and postoperative pneumocephalus

**Figure 5:**
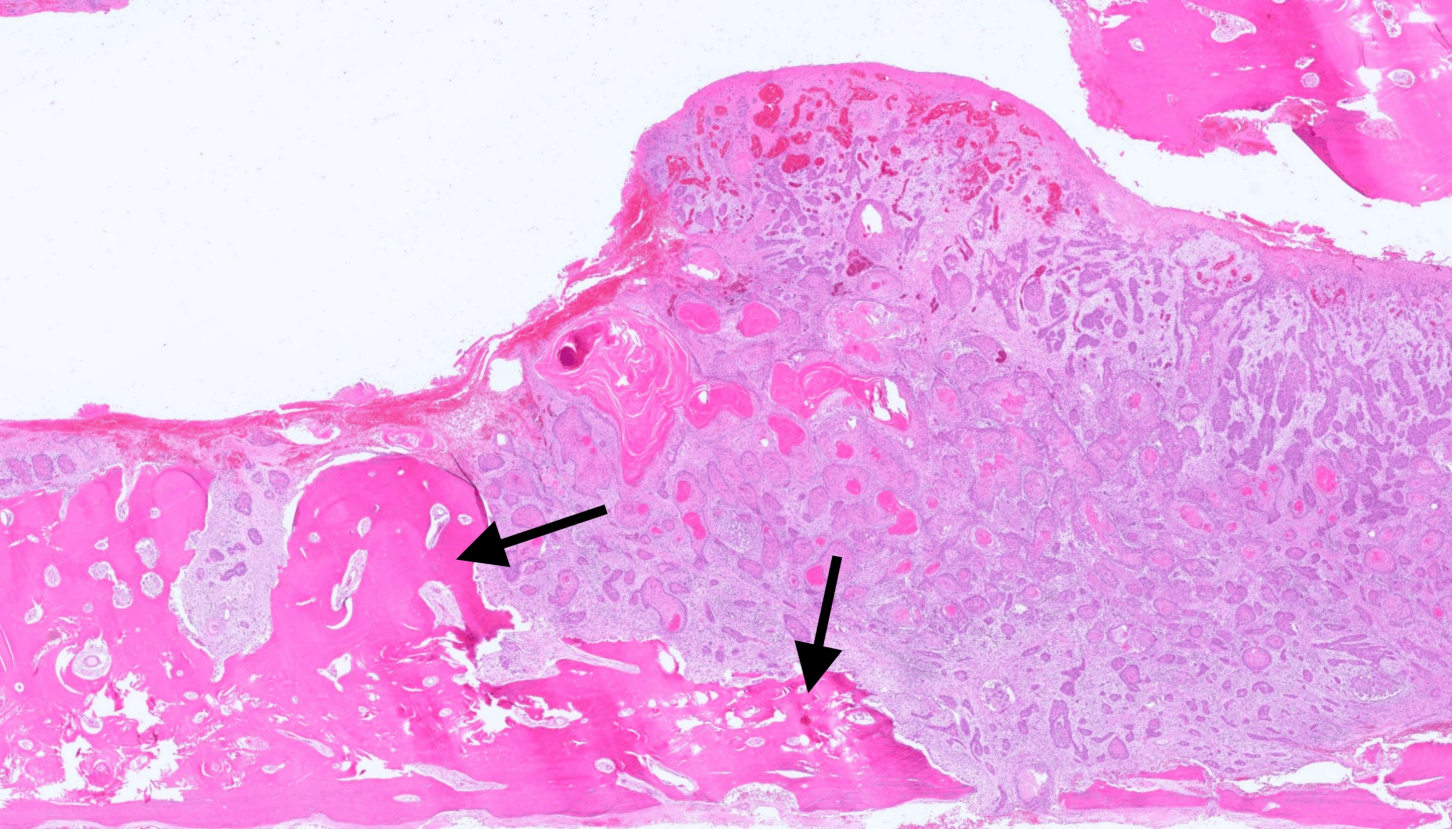
Low magnification hematoxylin and eosin staining showing cranial bone and dura mater invasion (arrow) by squamous cell carcinoma

**Figure 6:**
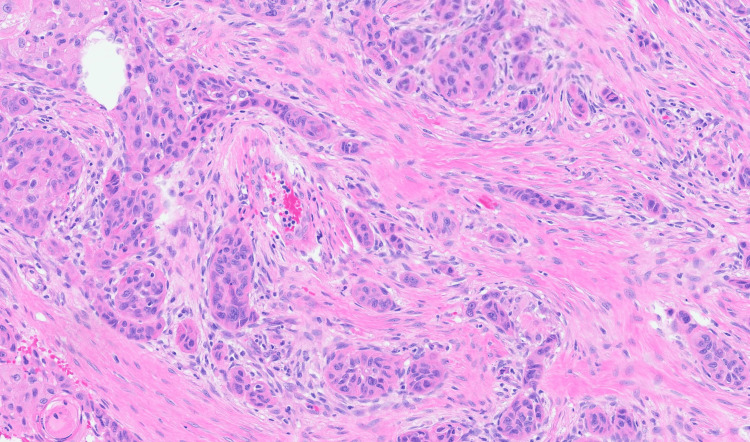
High magnification hematoxylin and eosin staining showing dural invasion

The multidisciplinary tumor board review recommended surgical intervention involving craniotomy with bone flap resection. The surgical team comprised neurosurgery, plastic surgery, and otolaryngology. Reconstruction was achieved through the placement of a free right latissimus dorsi musculocutaneous flap with microvascular anastomosis to cover the scalp defect, application of a 30 x 11 cm split-thickness skin graft to the cranial defect (Figure 3), and placement of a 23 x 18 cm Suprathel skin substitute (PolyMedics Innovations GmbH, Germany) to the left lower extremity donor site. Postoperative imaging confirmed graft placement and resection of the malignancy (Figure 4). Postoperative pathology confirmed SCC with invasion into the cranial bone and dura mater (Figures 5, 6). Despite initial satisfactory graft integration, the patient subsequently returned with evidence of disease recurrence in the thorax within a year.

## Discussion

Our patient presented with a rare scalp cSCC, exhibiting extensive tumor growth and unique radiographic findings demonstrating transcalvarial extension with dural impingement. This case presents an opportunity to discuss the risk factors for the development of cSCC in HIV-positive patients and the socioeconomic barriers for receiving early intervention.

The patient exhibited multiple established risk factors for scalp cSCC development, including a history of immunocompromised status, fair complexion, male sex, advanced age, and chronic ultraviolet radiation exposure. While HIV-positive patients share conventional cSCC risk factors with the general population, several critical distinctions warrant consideration [7]. Immunosuppression is associated with a younger age at diagnosis and an increased risk of developing cSCC compared to immunocompetent individuals [8,9]. This dramatic elevation underscores the importance of enhanced dermatologic surveillance in HIV-positive patients, particularly those with additional predisposing factors.

Patients with HIV are at a greater risk of developing locally advanced or metastatic cSCC [9]. Silverberg et al. found that immunosuppression had a statistically significant increased risk for cSCC metastasis [4]. Established metastatic risk factors include invasion beyond subcutaneous tissue, Breslow thickness exceeding 2 millimeters, diameter greater than 20 millimeters, and perineural invasion. These features, when present in immunocompromised patients, necessitate prompt staging and treatment.

Prevention and early diagnosis of cSCC are key in HIV-positive patients, particularly those experiencing housing insecurity and an elevated risk of loss to follow-up. Monitoring HIV viral load and CD4 counts provides essential prognostic information, as markers of severe disease (CD4 counts <200 cells/mm3) have been associated with increased cSCC risk [10]. HIV patients, especially those with additional risk factors, are at increased risk of progression to advanced cSCC, including metastasis or extensive local advancement. Rarely, scalp cSCC can locally invade into the skull, and such invasion is associated with poor prognosis and the requirement of multidisciplinary care.

Despite this patient's high-risk tumor characteristics, no sites of metastasis were identified. This finding, while seemingly incongruent with expected disease behavior, aligns with similar cases in the literature [11,12]. We hypothesize that the tumor's local advancement into the skull became so severe and clinically significant that treatment was initiated out of necessity, potentially intervening before the tumor naturally progressed to metastatic disease. Delayed diagnosis, patient neglect, and social determinants of health are common factors in cases such as this.

This single-patient case report relies on existing literature rather than comparative cohorts, limiting the generalizability of its findings. Further studies are needed to better characterize risk factors and outcomes in similar, high-risk, immunocompromised populations.

## Conclusions

This case demonstrates the devastating consequences of compounding social determinants of health in vulnerable populations, particularly the unhoused. The patient’s history of medication loss leading to inconsistent antiretroviral adherence highlights a critical challenge among unhoused individuals with chronic conditions. The lack of secure storage directly contributed to this patient’s increased immunocompromised state, creating optimal conditions for malignant transformation. His limited access to preventive care and medical follow-up allowed a mildly painful papule to progress unmonitored for months, by which time it had already invaded the skull. Malnutrition further compromised his immune function and wound-healing capacity.

The markedly aggressive clinical progression of cSCC was illustrated in the context of immunosuppression, demonstrating the capacity for significant local tissue invasion when diagnosis is delayed due to health care access disparities. This case emphasizes the critical necessity for targeted public health interventions for vulnerable populations experiencing homelessness. Initiatives should include skin cancer screenings, distribution of sun-protective resources, establishment of secure medication storage systems, and support for chronic disease management. Addressing the root causes of delayed diagnosis through improved healthcare accessibility and social support systems is crucial for preventing similar devastating outcomes in vulnerable populations. Future research should focus on identifying modifiable risk factors and effective interventions to reduce diagnostic delays and improve cancer outcomes among unhoused, immunocompromised individuals.
